# Autophagic responses compensate mitochondrial impairments

**DOI:** 10.18632/aging.101292

**Published:** 2017-09-11

**Authors:** Vanessa Henkel, Verena Warnsmann, Heinz D. Osiewacz

**Affiliations:** Institute of Molecular Biosciences and Cluster of Excellence Frankfurt Macromolecular Complexes, Department of Biosciences, J. W. Goethe University, 60438 Frankfurt, Germany

**Keywords:** aging, mitochondrial quality control, general autophagy, mitophagy

Mitochondria are the “power plants” of eukaryotic cells involved cellular energy metabolism and lead the generation of most of the cellular “energy currency” adenosine triphosphate (ATP). In addition, they have other crucial functions including the control of programmed cell death, iron/sulfur cluster biogenesis and copper and calcium homeostasis. Mitochondrial dysfunction is deleterious and leads to degeneration, disease and aging. A number of individual pathways are active in keeping mitochondria functional over longer periods of time and thereby have a strong impact on lifespan. These mitochondrial quality control (mtQC) pathways occur at different molecular and cellular levels and are all limited in their capacity. They do not all work at the same time. Some of them are induced when others fail. Currently, the underlying molecular interaction of pathways and their regulation is only initially elucidated.

**Figure 1:**
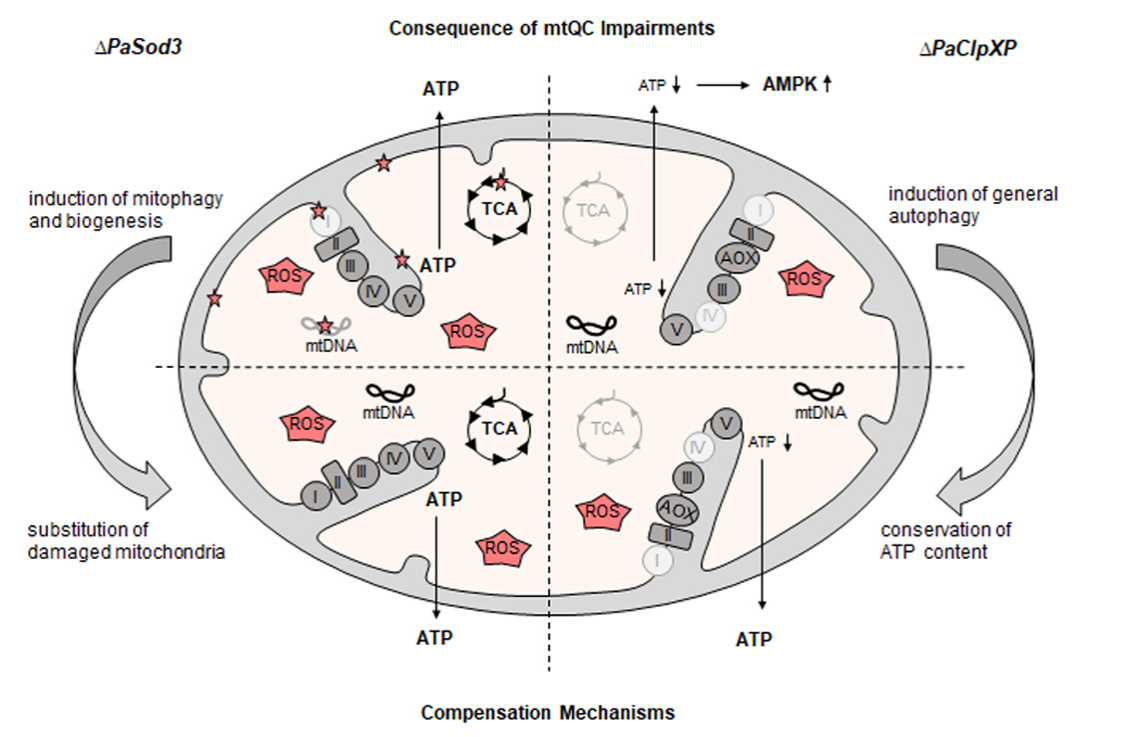
Compensation of mtQC impairments Loss of mitochondrial superoxide dismutase PaSOD3 leads to unspecific damage of all kinds of molecules (red stars) by ROS. This damage, as long as it is not excessive, does not significantly effect ATP generation. Within certain limits, the accumulation of damage can be compensated by induction of mitophagy and mitochondrial biogenesis, leading to a remodeling and/or substitution of damaged mitochondria and a wild-type-like lifespan. In contrast, ablation of the mitochondrial PaCLPXP leads to impaired mitochondrial respiration complexes and other enzymes of the energy metabolism (light grey). This impairment can be compensated by a response leading to the induction of alternative oxidase (AOX) and by upregulation of general autophagy and results in the conservation of a wild-type-like ATP content.

*Podospora anserina* is an experimental aging model in which different mtQC pathways have been shown to influence aging and lifespan [1, 2, 3]. For instance, a very basic pathway is involved in balancing levels of reactive oxygen species (ROS), which are both essential as well as harmful for biological systems. At low abundance they are involved in signaling and are necessary for proper organismic development. At increased levels they lead to unspecific damage of all kinds of biomolecules including proteins, lipids and nucleic acids (Figure 1) [4]. Another pathway is autophagy, the vacuolar degradation of cellular components. This pathway was found to act as a pro-survival or ‘longevity assurance’ pathway and increases during *P. anserina* wild type aging [5, 6].

In a recent study, we generated a mutant (∆*PaSod3*) in which the mitochondrial superoxide dismutase (PaSOD3) was ablated (Figure 1, left) and thereby the detoxification of the superoxide anion that is generated during respiration is inhibited. Counterintuitively, although impaired in this mtQC pathway, the mutant is characterized by the same lifespan as the wild-type strain [1, 3]. A detailed analysis of the deletion strain revealed that increased superoxide anion levels trigger the induction of mitophagy, a type of selective auto-phagy of mitochondria, as a compensatory mechanism of impaired mitochondrial ROS scavenging [3]. It appears that the organism can overcome the unspecific molecular impairments by ROS via an efficient degra-dation of damaged mitochondria and their remodeling and replacement by mitochondrial biogenesis.

In a further study, another component of the mtQC system was investigated (Figure 1, right), the mito-chondrial matrix protease complex PaCLPXP [2, 7]. Consisting of the CLPP serine protease and the CLPX chaperone that delivers specific substrates to the protease, the CLPXP protein complex is involved in degradation of proteins belonging to fundamental mitochondrial metabolic pathways [7]. The two single mutants, as well as the ∆*PaClpXP* double mutant are characterized by an unexpected lifespan extension [2]. Again, these results suggest the induction of similar compensatory mechanisms as in ∆*PaSod3*. However, unlike in ∆*PaSod3*, mitophagy is not induced in ∆*PaClpXP*, although also in this case mitochondrial function is impaired. Instead, general (bulk) autophagy, the non-selective degradation of cytoplasmic portions by autophagy is induced.

The data of the two studies identify a flexible context-dependent autophagic response and the induction of different forms of autophagy. In the ∆*PaSod3* mutant mitochondrial function does not result from a total block of a specific pathway (e.g., respiration). As long as the damage remains low, efficient remodeling of damaged mitochondria is sufficient to compensate the impaired function. In the ∆*PaClpXP* mutants induction of mitophagy is not the adequate response. In these mutants the whole mitochondrial energy metabolism is affected due to the function of the CLPXP complex to adapt the energy metabolism in response to the cellular needs and not in removal of damaged proteins. Ablation of this complex therefore leads to the deregulation of the mitochondrial metabolism and a subsequent adaptation via the induction of general autophagy (Figure 1). Cellular sensing of nutrient status by adenosine monophosphate-activated protein kinase (AMPK) is known to conserve of ATP levels by modulation of ATP consuming processes like protein biosynthesis. This scenario is supported by the observation that the ATP content of the ∆*PaClpXP* mutants appears wild-type-like, and strongly depends on functional autophagy machinery. The induction of general autophagy in ∆*PaClpXP* mutants thus resembles a situation after nutrient starvation. The molecular details involved in the induction of this response remain to be unraveled.

Overall, from recent analysis of the role of different mtQC pathways revealed unexpected and counter-intuitive results that identifies crosstalks between different pathways and revealed a context-dependent differential induction of different forms of autophagy. Impairments in individual mtQC pathways do not always result in the induction of mitophagy although in these cases mitochondria are affected in their function. The induced mitochondrial autophagic responses depend on the kind and level of mitochondrial impairments (Figure 1). Organisms seem to be able to differentiate between specific impairments and un-specific damage to induce efficient compensatory mechanisms.
